# Endogenous relapse and exogenous reinfection in recurrent pulmonary tuberculosis: A retrospective study revealed by whole genome sequencing

**DOI:** 10.3389/fmicb.2023.1115295

**Published:** 2023-02-17

**Authors:** Wencong He, Yunhong Tan, Zexuan Song, Binbin Liu, Yiting Wang, Ping He, Hui Xia, Fei Huang, Chunfa Liu, Huiwen Zheng, Shaojun Pei, Dongxin Liu, Aijing Ma, Xiaolong Cao, Bing Zhao, Xichao Ou, Shengfen Wang, Yanlin Zhao

**Affiliations:** ^1^National Institute for Communicable Disease Control and Prevention, Chinese Center for Disease Control and Prevention, Beijing, China; ^2^Hunan Provincial Chest Hospital, Tuberculosis Control Institution of Hunan Province, Changsha, Hunan, China; ^3^National Tuberculosis Reference Laboratory, Chinese Center for Disease Control and Prevention, Beijing, China; ^4^Laboratory of Respiratory Diseases, Beijing Key Laboratory of Pediatric Respiratory Infection Diseases, Beijing Pediatric Research Institute, Beijing Children’s Hospital, Capital Medical University, Beijing, China; ^5^School of Public Health, Peking University, Beijing, China; ^6^Shenzhen Third People’s Hospital, Shenzhen, China

**Keywords:** tuberculosis, recurrence, relapse, reinfection, whole genome sequencing

## Abstract

**Background:**

Tuberculosis may reoccur due to reinfection or relapse after initially successful treatment. Distinguishing the cause of TB recurrence is crucial to guide TB control and treatment. This study aimed to investigate the source of TB recurrence and risk factors related to relapse in Hunan province, a high TB burden region in southern China.

**Methods:**

A population-based retrospective study was conducted on all culture-positive TB cases in Hunan province, China from 2013 to 2020. Phenotypic drug susceptibility testing and whole-genome sequencing were used to detect drug resistance and distinguish between relapse and reinfection. Pearson chi-square test and Fisher exact test were applied to compare differences in categorical variables between relapse and reinfection. The Kaplan–Meier curve was generated in R studio (4.0.4) to describe and compare the time to recurrence between different groups. *p* < 0.05 was considered statistically significant.

**Results:**

Of 36 recurrent events, 27 (75.0%, 27/36) paired isolates were caused by relapse, and reinfection accounted for 25.0% (9/36) of recurrent cases. No significant difference in characteristics was observed between relapse and reinfection (all *p* > 0.05). In addition, TB relapse occurs earlier in patients of Tu ethnicity compared to patients of Han ethnicity (*p* < 0.0001), whereas no significant differences in the time interval to relapse were noted in other groups. Moreover, 83.3% (30/36) of TB recurrence occurred within 3 years. Overall, these recurrent TB isolates were predominantly pan-susceptible strains (71.0%, 49/69), followed by DR-TB (17.4%, 12/69) and MDR-TB (11.6%, 8/69), with mutations mainly in codon 450 of the *rpoB* gene and codon 315 of the *katG* gene. 11.1% (3/27) of relapse cases had acquired new resistance during treatment, with fluoroquinolone resistance occurring most frequently (7.4%, 2/27), both with mutations in codon 94 of *gyrA*.

**Conclusion:**

Endogenous relapse is the main mechanism leading to TB recurrences in Hunan province. Given that TB recurrences can occur more than 4 years after treatment completion, it is necessary to extend the post-treatment follow-up period to achieve better management of TB patients. Moreover, the relatively high frequency of fluoroquinolone resistance in the second episode of relapse suggests that fluoroquinolones should be used with caution when treating TB cases with relapse, preferably guided by DST results.

## 1. Introduction

Tuberculosis (TB) remains a major global public health issue, with an estimated 10.0 million new cases and more than 1.2 million deaths from TB worldwide in 2019 [World Health Organization (WHO), 2020]. Although most TB patients can be cured after the introduction of a standard combination of chemotherapy, some patients who complete an appropriate course of treatment still experience a subsequent episode, or TB recurrence (Zong et al., 2018). Patients with recurrent TB often require longer rounds of treatment with more toxic drugs, which reduces the success of treatment, leads to further transmission of *Mycobacterium tuberculosis* (MTB), and increases the burden of TB (Liu et al., 2020).

Recurrence of TB can be caused by relapse, also known as endogenous reactivation of the initial infection, or by exogenous reinfection with new MTB strains (Ruan et al., 2022). The proper discrimination between relapse and reinfection is essential for adjusting TB control measures. High relapse rates indicate inadequate TB treatment, whereas high rates of reinfection reveal poor TB cases management with many missed TB cases circulating in the community (Folkvardsen et al., 2020; Du et al., 2021).

The advent of molecular genotyping techniques for MTB has made it possible to assess the magnitude of endogenous relapse versus exogenous reinfection (Bandera et al., 2001; Lambert et al., 2003). These genomic-based typing methods include IS6110 fingerprinting, mycobacterial interspersed repetitive unit-variable number of tandem repeat (MIRU-VNTR), spoligotyping, and whole-genome-sequencing (WGS) (Barbier and Wirth, 2016). However, different genotyping methods often affect the reinfection rate due to different resolutions (Jagielski et al., 2016). Compared to traditional genotyping methods, WGS based on the full-genome of MTB strains has the distinct advantage by allowing the discrimination of MTBC strains at the highest resolution and simultaneously enabling detailed resistance predictions for almost all drugs (Roetzer et al., 2013; Walker et al., 2015).

Despite tremendous progress in TB control, China still has the second-highest TB burden worldwide [World Health Organization (WHO), 2020]. In addition, the presence of TB recurrence can further increase the burden of TB. A better understanding of the sources of recurrent TB and its related risk factors is essential for targeted interventions and for reducing the frequency of TB (Shen et al., 2017). However, limited efforts have been made to identify the major cause of TB recurrence in China, particularly in Hunan province, which has one of the highest TB burdens in China, with an estimated annual TB incidence of 94 cases per 100,000 population (He et al., 2022). To address this concern, we conducted a retrospective study among recurrent TB cases from five counties in Hunan province. We used WGS to determine whether TB recurrence was mainly caused by reinfection or relapse. We performed phenotypic drug susceptibility testing (DST) to compare *in vitro* DST results between the first and second TB episodes. We also collected demographic information and clinical characteristics of recurrent TB cases to analyze risk factors associated with reinfection and relapse.

## 2. Materials and methods

### 2.1. Study population

This retrospective study was conducted based on five DRS (drug resistance surveillance) sites (5 counties: Hecheng, Yongshun, Qidong, Taojiang, and Leiyang) in Hunan province, which were established according to the first national survey of drug resistance in China (Zhao et al., 2012). In these five counties, all suspected pulmonary TB cases from general hospitals or health centers are referred to local designated TB hospitals for confirmed diagnosis and treatment. All TB cases aged 15 years or older with bacteriologically confirmed (sputum-smear positive or culture positive) by local designated TB hospitals or clinics between January 1, 2013 to December 31, 2020 were included in this study. Positive sputum samples were cultured and isolated on Lowenstein-Jensen medium at the county-level and then sent to National Tuberculosis Reference Laboratory (NTRL). Information on these TB cases, including demographic characteristics and medical records, is collected at the time of patients’ visits and stored electronically in the National Tuberculosis Information Management System (TBIMS). To identify recurrent TB cases, the medical records of TB patients diagnosed between 2013 and 2020 were extracted from TBIMS on June 30, 2022 and collated using the method described previously (Shen et al., 2017). TB cases with any of the followings were excluded from the further study: (1) unsuccessful treatment outcomes of their initial TB episode (e.g., lose to follow-up, death, treatment failure, etc.); (2) less than 6 months of the recurrent interval (the time interval between the recorded end date of the treatment and the date of the re-diagnosis of active TB); (3) strains with subculture failure or contamination; (4) failed extraction of DNA or WGS errors.

### 2.2. Drug susceptibility testing

All MTB strains isolated from recurrent TB cases were previously stored in 7H9 medium containing 25% glycerin at–80°C refrigerator, and then were thawed and re-cultured on L-J medium for further study. MTB isolates in the logarithmic phase were subjected to drug susceptibility testing against rifampicin, isoniazid, ethambutol, streptomycin, ofloxacin, moxifloxacin, kanamycin, and amikacin using MYCOTB plate (Thermo Fisher Scientific, United States). Previous studies have demonstrated the good accuracy and reproducibility of the MYCOTB plate, which can be used as an alternative method for DST (Xia et al., 2017; Wu et al., 2019). All procedures were performed by trained staff at the national TB reference laboratory of China, as described elsewhere (He et al., 2022). H37Rv (ATCC 27294) was used as pan-susceptible control in each batch of DST. The concentration ranges and cut-off values for determining resistance or sensitivity for each drug used in this study were depicted previously (He et al., 2022). All DSTs were conducted twice to ensure the accuracy of DST results.

### 2.3. DNA extraction and sequencing

MTB strains were scraped from L-J solid slants, and genomic DNA was obtained from isolates with the cetyltrimethylammonium bromide (CTAB) method as described previously (Shao et al., 2021). The quality and concentration of genomic DNA were assessed by NanoDrop 2000c spectrophotometer (Thermo Fisher Scientific, USA) and Qubit 2.0 fluorometer (Invitrogen, Thermo Fisher Scientific, USA), respectively. Whole genome sequencing was performed by Annoroad Gene Technology company (Beijing, China) using Illumina Hiseq X10 (Illumina, Inc.) with 2 × 150 paired-end (PE) strategies.

### 2.4. Phylogenetic analysis

In brief, the quality control of raw reads was examined by FastQC (v0.11.9),^1^ and reads were filtered with Trimmomatic (v 0.38) using default values and minimum Phred Quality score of 20 (Bolger et al., 2014). Retained paired-end reads were mapped to the reference genome H37Rv (GenBank accession NC_000962.3) using BWA-MEM software (v. 0.7.17) (Freschi et al., 2021). Variants information including single nucleotide polymorphisms (SNPs) and small insertion/deletions (Indel) were detected using SAMtools (v1.3.1) and GATK (v.3.8.0) (He et al., 2022). The variants that met the following criteria were kept for further analysis: minimum coverage depth of 10X, Q20 minimum quality score for each variant, and more than 75% allele frequency (He et al., 2022).

SNPs located in repeating regions of the genome such as PE/PPE/PGRs genes, phage sequence, insertions, and mobile elements were excluded. The remaining SNPs in each isolate were pooled into a sequence based on the position, and SNP positions present in at least 95% of isolates were integrated into a sequence alignment (Liu et al., 2022). The maximum likelihood trees were constructed using a general time reversible model in MEGA-X (v.10.1.8) with bootstraps of 1,000 replicates (He et al., 2022). The phylogenetic tree was visualized and annotated using iTOL.^2^ Snp-dists (v.0.8.2) was used to calculate the SNP distance between pairs of isolates. QuantTB (v. 1.01)^3^ was used to identify mixed infection of MTB (Anyansi et al., 2020).

### 2.5. Lineage and genotypic drug resistance prediction

Fast-lineage-caller package (v.3.2)^4^ was used to call lineage and sub-lineage information of *M.* tuberculosis. TB Profiler (v.3.0.8)^5^ was used to predict genotypic drug susceptibility.

### 2.6. Statistical analysis

Pearson chi-square test and Fisher exact test were used to compare differences in categorical variables between relapse and reinfection. The Kaplan–Meier curve was generated in R studio (4.0.4) to describe and compare the time to recurrence between different groups. All statistical analysis was performed in the SPSS version 18.0 software (SPSS Inc., Chicago, Illinois). *p* < 0.05 was considered statistically significant.

### 2.7. Definition

TB recurrence was defined as a patient who was cured or completed treatment during the most recent course of treatment and then was re-diagnosed with a new TB episode [World Health Organization (WHO), 2013]. Reinfection was defined as a recurrent disease episode caused by a new TB strain with a genetic distance of more than 12 SNPs compared with the strain that caused the original episode. Relapse was defined as a genetic distance of 12 or fewer SNPs between paired strains isolated from two episodes in TB recurrence (Li et al., 2022). The recurrent interval was defined as the time interval between the recorded end date of the initial TB treatment and the date of the re-diagnosis of active TB (Ruan et al., 2022). Based on the phenotypic drug susceptibility testing, Pan-Susceptible was defined as MTB strains that were susceptible to all anti-TB drugs tested in this study (including rifampicin, isoniazid, ethambutol, streptomycin, moxifloxacin, ofloxacin, kanamycin and amikacin), whereas Drug-resistant was defined as MTB strains that were resistant to at least one of these anti-TB drugs but not include the concurrent resistance to rifampicin and isoniazid. MDR-TB was defined as MTB resistance to at least isoniazid and rifampicin. Pre-XDR-TB was defined as MDR-TB with additional resistance to any fluoroquinolones (moxifloxacin or ofloxacin) or any second-line injectable drugs (amikacin or kanamycin), but not both. XDR-TB was defined as MDR-TB with additional resistance to any fluoroquinolones and any second-line injectable drugs.

## 3. Results

### 3.1. Description of the study population

**Figure 1:**
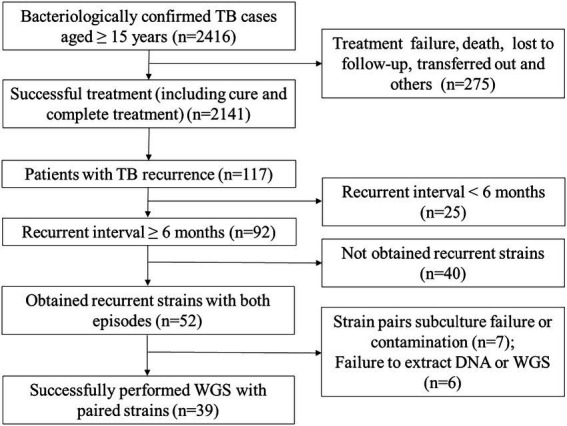
Flowchart of recurrent TB cases included and excluded from the study.

A total of 2,416 bacteriologically confirmed TB cases aged 15 years or older were collected between Jan. 2013 and Dec. 2020. Of which, 88.6% (2141/2416) cases were successfully treated, while 275 (11.4%) patients experienced treatment failure, loss to follow-up, treatment interruption, adverse reactions, or death. Overall, 117 (5.5%, 117/2141) successfully treated cases that experienced TB recurrences, 25 recurrent TB cases were excluded due to their recurrent interval being less than 6 months, and finally, 92 recurrent TB cases were included in further analysis. Among them, 56.5% (52/92) had recurrent strains with both episodes. After excluding subculture failure or contamination of any paired isolates (*n* = 7) and failure to extract DNA or WGS (n = 6). Finally, 39 recurrent TB patients with paired strains were enrolled in the final analysis. Of these, one patient had a third episode during the study period, for a total of 79 MTB isolates and 41 recurrent events (Figure 1).

### 3.2. Patient characteristics

The characteristics of recurrent TB cases at their primary episode were described below. Among the 39 recurrent TB cases included in this study, the median and mean age of patients was 54.0 [interquartile range (IQR), 45.0–65.0] and 54.4 ± 12.6 years old. The majority of patients were male (84.6%, 33/39) while 15.4% (6/39) were female. Almost 90% (89.7%, 35/39) of patients were farmers. In terms of treatment history, new cases accounted for 94.9% (37/39) of the total. 7.7% (3/39) and 12.8% (5/39) of recurrent TB cases had complications of hepatitis B and diabetes, respectively. The chest X-ray showed that 30.8% (12/39) of patients had cavitation in the first episode of TB. As for HIV status, 12 (30.8%, 12/39) patients were HIV-negative, while 27 (69.2%, 27/39) patients had unknown HIV infection status.

### 3.3. TB relapse and reinfection identified by SNP distance

**Figure 2:**
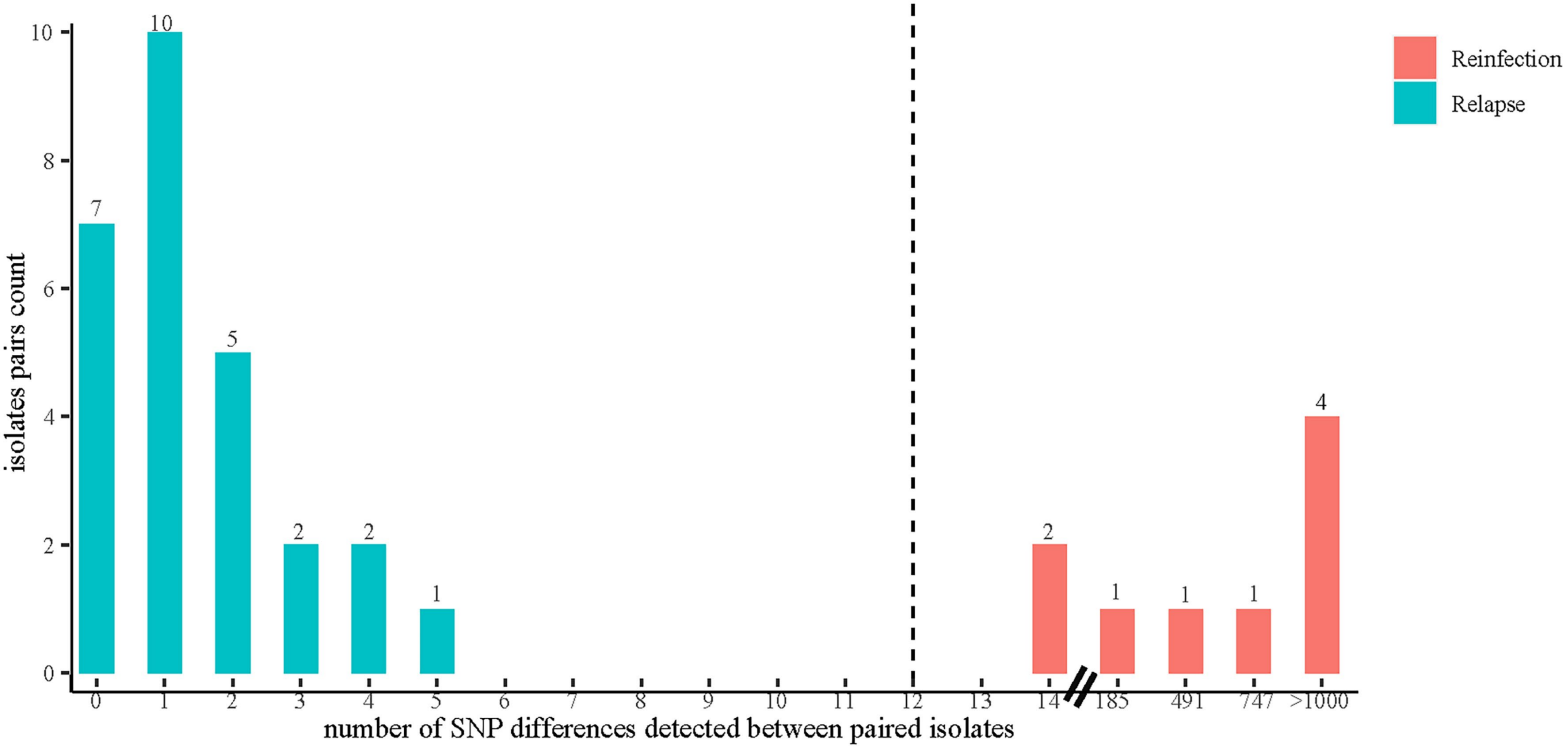
The distribution of SNP differences between paired isolates. Reinfection was defined as a recurrent disease episode caused by a new TB strain with a genetic distance of more than 12 SNPs compared with the strain that caused the original episode. Relapse was defined as a genetic distance of 12 or fewer SNPs between paired strains isolated from two episodes in TB recurrence. The SNP differences between paired isolates were calculated by using Snp-dists (v.0.8.2).

The whole genome sequencing data of 79 MTB strains collected from 39 recurrent TB cases were first analyzed to determine the presence of mixed infections. Five recurrent TB cases (7 strains of mixed infections in total) were excluded from further analysis because any of their paired strains were identified as having at least two different strains. The paired SNP distances were calculated on the remaining 34 recurrent TB cases, including one patient (patient 15) with three TB episodes (patient 15–1, 15–2, 15–3), thus involving a total of 69 MTB strains and 36 recurrent events (Supplementary Figure S1). Identical genotypes were defined as strains that differed by no more than 12 SNPs (Li et al., 2022). Of 36 recurrent events, 27 (75.0%, 27/36) paired isolates (patient 1, 2, 3, 4, 5, 7, 8, 9, 11, 12, 13, 14, 15[1–3], 16, 20, 21, 22, 23, 24, 25, 26, 27, 28, 30, 31, 32 and 33) had 5 or fewer SNP differences (Supplementary Figure S1), indicating relapse. Nine (25.0%, 9/36) paired isolates (patient 6, 10, 15[1–2], 15[2–3], 17, 18, 19, 29, and 34) differed by >12 SNPs, suggesting exogenous reinfection with a new strain of MTB (Supplementary Figure S1). Comparison with SNP differences was also shown in Figure 2, identifying two major groups: paired isolates from relapsed cases had five or fewer SNP differences, whereas paired strains from reinfected cases had a dramatically higher number of SNP differences (range 185–1,074) except for 2 paired isolates (patient 17 and 18) with SNP differences of 14 (Supplementary Figure S1; Figure 2).

### 3.4. Phylogenetic reconstructions and drug-resistant profile

**Figure 3:**
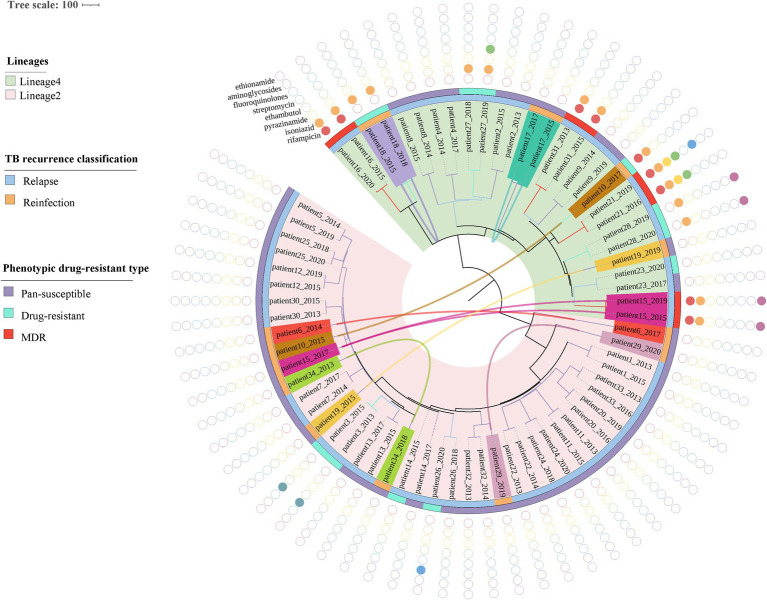
Phylogenetic tree and drug-resistant profile of 69 MTB strains from 34 recurrent patients. Inner band indicates TB recurrence classification (reinfection represents strain pairs differences >12 SNPs, whereas relapse represents strain pairs differences ≤12 SNPs) and the outer band suggests phenotypic drug-resistant type (see legend). Solid circles indicate genetic drug resistance detected by TB-profiler. Reinfected patients are highlighted with different colors and curves connecting patients’ samples in the phylogeny indicating paired strains isolated from the same patient.

The phylogenetic tree was constructed based on 6,847 high-quality SNPs (Figure 3). Fast-lineage-caller analysis showed that the majority (59.4%, 41/69) of recurrent TB isolates were lineage 2, and 40.6% (28/69) were lineage 4. All the isolate pairs from relapse cases were close together on the tree, whereas almost the reinfected isolate pairs appeared quite divergent on the tree (marked in different colors) (Figure 3). We also analyzed the community transmission of these recurrent TB cases, as demonstrated in Figure 3, TB strains collected from different individuals did not show high sequence similarity. Among these recurrent TB isolates, pan-susceptible predominated, accounting for 71.0% (49/69), with only 8 (11.6%, 8/69) and 12 (17.4%, 12/69) were identified as MDR-TB and DR-TB, respectively. To rationalize these phenotypic drug-resistance, genetic mutations were predicted based on WGS data. A total of 9 recurrent TB strains were identified as genomic MDR-TB, mainly with mutations in codon 450 of the *rpoB* gene and codon 315 of the *katG* gene (Table 1). Moreover, 2 strains had detectable drug-resistant mutations to four first-line anti-TB drugs (rifampicin, isoniazid, pyrazinamide, and ethambutol) simultaneously. We further compared the drug-resistant profiles between paired isolates to clarify the development of acquired resistance during treatment. As shown in Figure 3, three relapse cases had acquired new resistance during treatment: two (patient 21 and 26) to fluoroquinolones were both due to mutations in codon 94 of *gyrA* and one (patient 27) to ethambutol due to a mutation in codon 406 of *embB*, resulting in amino acid substitution from Gly to Asp (Table 1). Of note, the strain from patient 21, which was MDR with additional resistance to ethambutol and pyrazinamide in the first isolate, had progressed to pre-XDR in their second relapsed isolate. Interestingly, one patient (patient 15) had three TB episodes between 2015 and 2019, of which the first and third episodes isolated the identical TB strain, both MDR-TB, while the new strain isolated from the second was pan-susceptible (Figure 3, Table 1). In addition, one patient (patient 10) was initially infected with a pan-susceptible strain and subsequently reinfected with a new strain that harbored gene mutations related to rifampin and isoniazid resistance (Table 1).

**Table 1:** Genetic mutations related to drug-resistance identified in the first and second episodes of recurrent TB isolates.

Recurrence classification	Patient ID	Drug	Episode 1	Episode 2
Relapse	Patient 3	SM	*rpsL* K43R	*rpsL* K43R
	Patient 15[1–3]	RIF	*rpoB* S450L	*rpoB* S450L
		INH	*fabG1*_c.-15C > T	*fabG1*_c.-15C > T
		ETH	*fabG1*_c.-15C > T	*fabG1*_c.-15C > T
	Patinet 16	RIF	*rpoB* H445Y & D435F	*rpoB* Q432P
		INH	*katG* S315N	*katG* S315N
	Patient 21	RIF	*rpoB* S450W	*rpoB* S450W
		INH	*katG* S315N	*katG* S315N
		EMB	*embB* D1024N	*embB* D1024N
		PZA	*pncA* L85R	*pncA* L85R
		FQs	WT	*gyrA* D94Y, *gyrB* Glu501D
	Patient 26	FQs	WT	gyrA D94G & A90V
	Patient 27	INH	*katG* Q461P & D94G	*katG* Q461P
		EMB	WT	*embB* G406D
	Patient 28	INH	*fabG1_*c.-15C > T	*fabG1*_c.-15C > T
		ETH	*fabG1_*c.-15C > T	*fabG1*_c.-15C > T
	Patient 31	RIF	*rpoB* D435V & H445Y	*rpoB* D435V
		INH	*katG* S315N	*katG* S315N
Reinfection	Patient 10	RIF	WT	*rpoB* L430P
		INH	WT	*katG* S315T
	Patient 15[1–2]	RIF	*rpoB* S450L	WT
		INH	*fabG1*_c.-15C > T	WT
		ETH	*fabG1*_c.-15C > T	WT
	Patient 15[2–3]	RIF	WT	*rpoB* S450L
		INH	WT	*fabG1*_c.-15C > T
		ETH	WT	*fabG1_*c.-15C > T
	Patient 18	INH	*katG*_c.-7G > A	*katG*_c.-7G > A

RIF, rifampicin; INH, isoniazid; EMB, ethambutol; PZA, pyrazinamide; SM, streptomycin; FQs, fluoroquinolones; ETH, ethionamide; WT, wild type.

### 3.5. Comparison of the characteristics between relapse and reinfection

**Figure 4:**
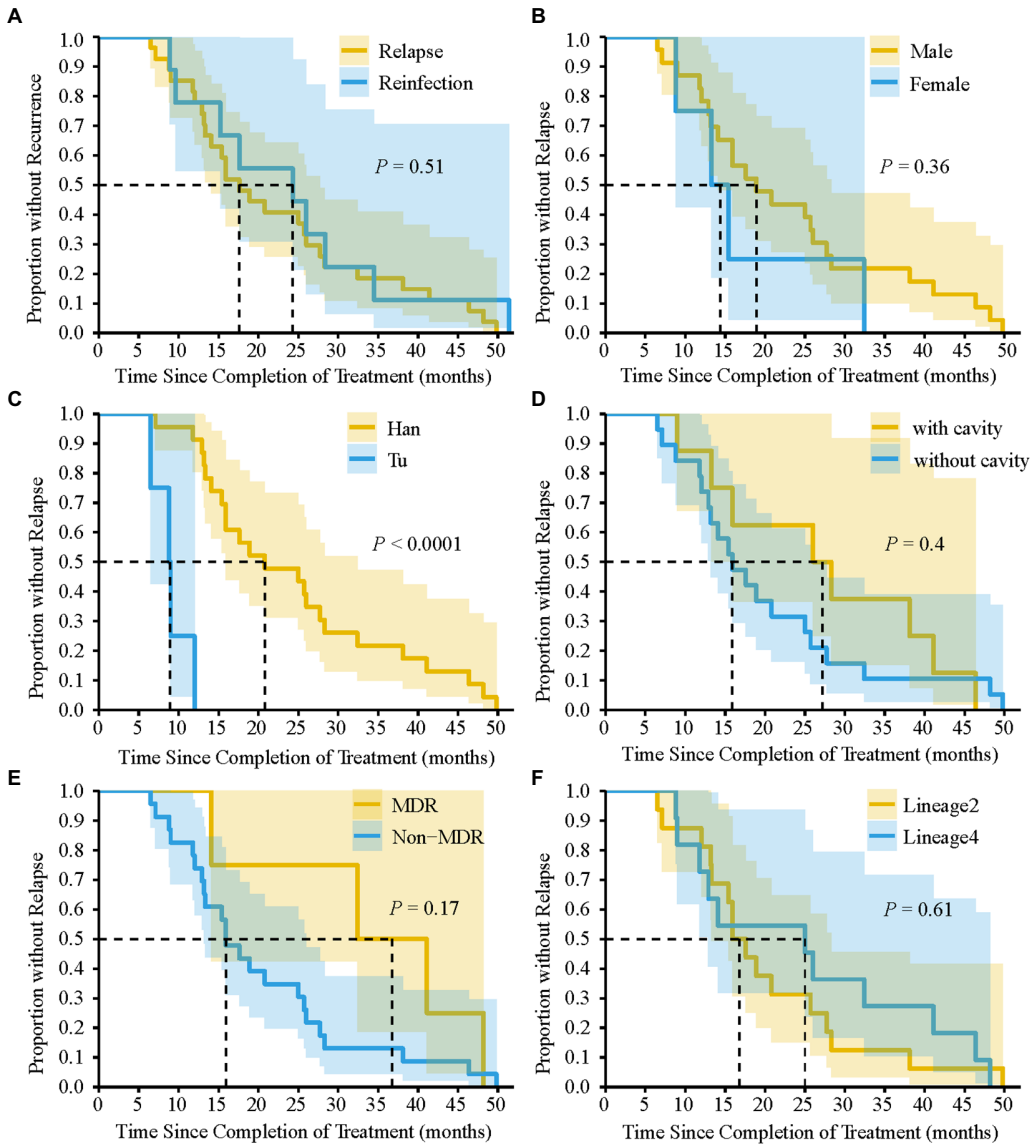
Kaplan–Meier survival estimates for TB recurrence. Comparison of the time interval required for relapse and reinfection **(A)**. The time interval required for relapse to occur by gender **(B)**, nationality **(C)**, pulmonary cavity **(D)**, MDR-TB **(E)**, and Lineage of TB strains **(F)**. dashed line indicate that the time interval required for 50% of TB recurrence or relapse to occur. *p*-value was calculated by Log-rank test.

We analyzed the differences in the characteristics between TB relapse and reinfection. As summarized in Table 2, all these demographic factors and clinical characteristics of patients, such as gender, age, occupation, and comorbidities et al., as well as genetic background and drug-resistant type of strains, had no significant effect on the proportion of TB relapse (all *p* > 0.05). In addition, more than 80% of TB recurrence occurred within three years after completion of treatment for the index episode (Table 2). The median of the recurrent time interval to relapse was 17.6 months (IQR, 12.9–28.3 months) compared with 24.3 months (IQR, 12.9–31.5 months) for reinfection cases, and there was no significant association between relapse and earlier recurrence (*p* = 0.51) (Figure 4A). We further assessed the time interval to relapse stratified by gender, nationality, pulmonary cavity, strain drug-resistant type, and genetic background. As shown in Figure 4, TB relapse occurs earlier in patients of Tu ethnicity compared to patients of Han ethnicity (*p* < 0.0001), whereas no significant differences in the time interval to relapse were noted in other groups.

**Table 2:** Initial episode characteristics in cases with TB relapse and reinfection.

Characteristic	Total	Endogenous relapse	Exogenous reinfection	*p*-value
	*N* (Column %)	*n* (Row %)	*n* (Row %)	
**Sex**				0.627*
Male	30 (83.3)	23 (76.7)	7 (23.3)	
Female	6 (16.7)	4 (66.7)	2 (33.3)	
**Age^#^**				0.869^a^
Median (IQR)	51.5 (45.0–63.8)	52.0 (42.0–63.0)	50.0 (47.0–65.5)	
**Nationality**				>0.999*
Han	31 (86.1)	23 (74.2)	8 (25.8)	
Tu	5 (13.9)	4 (80.0)	1 (20.0)	
**Occupation**				>0.999*
Farmer	32 (88.9)	24 (75.0)	8 (25.0)	
Non-Farmer	4 (11.1)	3 (75.0)	1 (25.0)	
**Pulmonary cavity**				0.235*
Yes	13 (36.1)	8 (61.5)	5 (38.5)	
No	23 (63.9)	19 (82.6)	4 (17.4)	
**Diabetes**				0.255*
Yes	4 (11.1)	2 (50.0)	2 (50.0)	
No	32 (88.9)	25 (78.1)	7 (21.9)	
**Hepatitis B**				0.558*
Yes	3 (8.3)	3 (100.0)	0 (0.0)	
No	33 (91.7)	24 (72.7)	9 (27.3)	
**Time of recurrent episode (after treatment completion of initial episode)**				0.651^b^
1st year	8 (22.2)	6 (75.0)	2 (25.0)	
2nd year	12 (33.3)	10 (83.3)	2 (16.7)	
3rd year	10 (27.8)	6 (60.0)	4 (40.0)	
4th year or later	6 (16.7)	5 (83.3)	1 (16.7)	
**Lineages**				>0.999*
Lineage 2	22 (61.1)	16 (72.7)	6 (27.3)	
Lineage 4	14 (38.9)	11 (78.6)	3 (21.4)	
**Drug-resistant type**				>0.999*
Pan-susceptible	26 (72.2)	19 (70.4)	7 (77.7)	
Drug-resistant	5 (13.9)	4 (14.8)	1 (11.1)	
MDR	5 (13.9)	4 (14.8)	1 (11.1)	

^#^Age was presented as median and inter-quartile range (IQR); *Value by Fisher’s exact test; ^a^Value by Mann–Whitney U test; ^b^Chi-square test for trend.

### 3.6. SNPs in relapse isolate

Of 39 SNPs and small indels (insertion–deletion) identified between the relapse pairs, 23 were non-synonymous polymorphisms. These mutations are located in genes encoding proteins with various functions, such as cell wall and cellular process, lipid metabolism, and information pathways (Supplementary Table S1). In three and four cases, these mutations were involved in drug-resistant related genes and growth advantage regions, respectively. We also identified 6 indels differences that result in frame-shifts within protein-coding regions, but all of these indels were located in non-essential regions (Supplementary Table S1).

## 4. Discussion

To our knowledge, this is the first longitudinal population-based study of sufficient duration to investigate TB recurrence using WGS in Hunan, China. The current study found a relatively high frequency of mixed infections among recurrent TB cases. After excluding patients with mixed infections, our study demonstrated that TB recurrence in Hunan province is mainly caused by endogenous reactivation of the initial infection (relapse), and reinfection accounted for a quarter of recurrent cases. In addition, our study found TB recurrence can occur even more than 4 years after treatment completion of the most recent episode, mainly within 3 years. Evidence of acquired resistance during treatment was also observed in this study, with fluoroquinolone resistance occurring most frequently.

Understanding the proportion of reinfection and relapse will help to implement better post-treatment follow-up and reduce TB burden. Unexpectedly high rates of reinfection suggest that reducing the risk of TB transmission is fundamental, while higher rates of relapse suggest that TB control should focus on improving the efficacy of the first-episode treatment regimen (Folkvardsen et al., 2020; Du et al., 2021). Numerous studies have shown that the proportion of TB recurrence due to exogenous reinfection varies by regions (Bandera et al., 2001; Verver et al., 2005; Zong et al., 2018; Liu et al., 2022). It is generally accepted that the proportion of reinfection in TB recurrence is higher in settings with a high prevalence of TB (Vega et al., 2021; Li et al., 2022), but there are exceptions (Shamputa et al., 2007). Studies of countries with low to moderate TB incidence found that the percentage of reinfection ranging from 10% in Switzerland to 33% in Spain (Schiroli et al., 2015), while reinfection was common in studies of high-burden countries, ranging from 23% in India to 68–77% in South Africa (Sahadevan et al., 1995; van Rie et al., 1999; Charalambous et al., 2008). Our study found that 25% of TB recurrence were attributed to reinfection, which was comparable to the proportion reported in Jiangsu (28.9%) (Liu et al., 2022), but much higher than that reported in Beijing (8.8%) (Du et al., 2021). Several reasons could be responsible for such variation of the percentage of reinfection. Firstly, the varied duration of follow-up would potentially affect the proportion of recurrence due to reactivation and reinfection (Liu et al., 2022). In general, relapse occurs earlier than reinfection, and if cases were followed up for an insufficient period, reinfections would not be captured (Vega et al., 2021), leading to a relatively lower proportion of reinfection. Secondly, different genomic-based typing methods, such as MIRU-VNTR, IS6110 fingerprinting, and whole genome sequencing, have different discriminatory power that can make a difference in the classification of TB recurrence (Shao et al., 2021). In addition, some of the patients’ complications could increase the risk from infection to disease, resulting in more reinfection cases (Lieberman et al., 2016). Moreover, transmission dynamics were also analyzed in our study and community transmission was not observed among these recurrent TB cases, which might due to transmission occurring in a broader population that was not included in our study population.

Mixed infections can complicate TB diagnosis and treatment, and it is also one of the potential confounders in distinguishing relapse from reinfection (Witney et al., 2017). To reduce the misclassification of recurrent TB cases, detection of mixed infection based on whole genome sequencing before determining the main source of TB recurrence is very essential. By using QuantTB, a method for identifying and quantifying individual MTB strains at high resolution (Anyansi et al., 2020), 7 of 79 (8.9%) isolates in this study were identified as mixed infections. Although the sampling and culture methods used in this study may lower the diversity of strains (Liu et al., 2020), a relatively high proportion of mixed infections were still detected, which warned of the urgent need for further studies to determine the prevalence of mixed infections in different settings and its impact on heterogeneous drug-resistance.

Of note, two patients’ pair (patient 17 and patient 18) of isolates in this study displayed 14 SNPs (SNP > 12) between two episodes and were therefore initially classified as reinfection. However, further analysis showed that the strain pairs were located next to each other on the phylogenetic tree and shared the same drug-resistant profile (Figure 2), suggesting that these two recurrent cases were likely caused by relapse. This would leave 7 recurrent cases with paired isolates differing by more than 180 SNPs that were clearly identified as the result of reinfection, indicating that 80.6% of TB recurrences were caused by relapse. The data here was supported by the findings of Walker and colleagues that the diversity between the initial and later isolates from relapsed patients does not generally exceed 14 SNPs, with most cases differing by less than five (Walker et al., 2013). Based on these results, it is reasonable to assume that strains with SNP differences slightly exceeding the thresholds (commonly 6 or 12 SNPs) used to define a cluster may occasionally belong to the same transmission chain and should be taken into account during the epidemiological investigation (Liu et al., 2020). More importantly, similar to previous studies, our study only found reinfections with large phylogenetic distances (range 185–1,074), but nothing at an intermediary level (Witney et al., 2017). This suggests that primary infection does not provide sufficient immune protection against genetically distant strains, which has important implications for future vaccine design (Bryant et al., 2013).

The emergence of drug resistance in relapsed TB weakens the effectiveness of subsequent treatment. In the present study, we found that the acquisition of resistance to fluoroquinolones was the most common during treatment, and this observation was further rationalized by genotypic resistance prediction based on whole-genome sequencing. Similar results have been reported elsewhere (Zong et al., 2018; Du et al., 2021). Although the exact cause of this phenomenon remains unclear, it can be partially explained by the abuse and misuse of fluoroquinolones. In China, because of their broad-spectrum antimicrobial activity, fluoroquinolones are always used as empirical treatment for suspected TB patients and various other types of infections (Du et al., 2021). Consequently, the selection pressure on MTB generated by residual drugs in the host allows the survival and accumulation of drug-resistant strains, resulting in strains with drug-resistance becoming the dominant population. Consistent with our findings, numerous previous studies have confirmed significantly increased prevalence of fluoroquinolones resistance in recent years in China (Xia et al., 2021; Mave et al., 2022). In addition, experimental data showed that fluoroquinolones activate the SOS response, which is likely to be associated with an elevated mutation rate. This may be another important factor contributing to the high frequency of fluoroquinolones resistance (Iacobino et al., 2021).

TB relapse was determined by a wide range of factors, such as socio-demographic and clinical features of TB cases, drug resistance and genetic background of the bacteria, and the disease burden of the study settings (Romanowski et al., 2019). Previous studies have shown that patients infected with Beijing genotype or isoniazid resistant strains were more susceptible to relapse (Hang et al., 2015; Thai et al., 2018). Besides, Romanowski et al. already found that despite poor predictive ability, cavitary disease and 2-month smear positivity could be used as markers for higher risk of relapse (Romanowski et al., 2019). However, in our current study, the relatively small sample size of recurrent TB cases limits our ability to detect significant difference between relapse and reinfection. To make follow-up for TB relapse more practical, future studies could identify socio-environmental and bio-medical factors associated with relapse by using modeling studies or genome-wide association analysis (GWAS), so these can be addressed or guide care after cure. Understanding the time interval distribution of recurrence is important for developing post-treatment control strategies and designing clinical trial studies (Marx et al., 2014). A meta-analysis reported that relapse occurred mainly in the first year after the end of treatment, while late recurrences tended to be reinfections (Romanowski et al., 2019). However, in our study, there was no significant difference in the time interval between relapse and reinfection. TB recurrences, whether caused by relapse or reinfection, occur predominantly within 3 years after completion of therapy. Therefore, for better management of TB patients in this region, we recommend that patients should be followed-up for at least 3 years after completion of therapy. Moreover, we further assessed the time interval to relapse stratified by gender, nationality, pulmonary cavity, et al. Despite the small sample size, a correlation was observed in the present study between Tu nationality and earlier relapse. Further study with an expanded sample size is needed to explore whether there is a genuine correlation between nationality and time interval to relapse.

A major strength of this study is that we conducted a retrospective study of sufficient duration by using whole-genome sequencing data of serial strains from recurrent TB patients, which allowed us to get a more accurate picture of the proportion of recurrence caused by reinfection after excluding mixed infections, as well as to understand the drug resistance acquired during treatment. We must acknowledge several limitations of this study. First, this study was based on routinely collected information and specimens. Some TB recurrent cases might be lost due to death or moving out of the region, which would reduce the accuracy of our results. Second, recurrent TB cases who were excluded from the final analysis due to subculture failure and contamination of any paired isolates may introduce selection bias into this study. Third, the relatively small sample size of drug-resistant TB strains restricted us from exploring the underlying mechanism of acquired drug resistance during treatment. Lastly, the HIV status of most recurrent TB cases in this study is unknown, but given the low prevalence of HIV in this area, we believe this is unlikely to introduce bias to the results of our study.

In conclusion, our data demonstrate that endogenous relapse is the main mechanism leading to TB recurrences in Hunan province. Additionally, our study found TB recurrence can occur even more than 4 years after treatment completion of the most recent episode, mainly within 3 years. Therefore, it is necessary to extend the post-treatment follow-up period to achieve better management of TB patients. Moreover, the relatively high frequency of fluoroquinolone-resistance in the second episode of relapse suggests that fluoroquinolones should be used with caution when treating TB cases with relapse, preferably guided by DST results.

## Data availability statement

The datasets presented in this study can be found in online repositories. The names of the repository/repositories and accession number(s) can be found in the article/Supplementary material.

## Ethics statement

Ethical approval was not provided for this study on human participants because National TB drug-resistant surveillance (DRS) was ethically approved by the Ethics Committee of Chinese Center for Disease Control and Prevention since the first national survey in 2007 (Zhao et al., 2012). Ethics approval of the present study was waived because all TB isolates used in this study were obtained from previous DRS routine work, and patient information was extracted from the previous database, no additional data and specimens were collected. Patients/participants provided their written informed consent at the time of their first visit to the designated TB clinics or centers.

## Author contributions

WH and YZ contributed to study design, data analysis, and manuscript writing. YT, ZS, BL, CL, HZ, DL, SP, and FH participated in study design, data collection, and analysis. YW, PH, AM, XC, and BZ conducted laboratory testing. HX, SW, and XO revised and polished the manuscript. All the authors have read the final version of the manuscript and have approved it.

## Funding

This work was supported by the National Key R&D Program (No. 2022YFC2305200) and Natural Science Foundation of Xinjiang Uygur Autonomous Region (No. 2022D01A115).

## Conflict of interest

The authors declare that the research was conducted in the absence of any commercial or financial relationships that could be construed as a potential conflict of interest.

## Publisher’s note

All claims expressed in this article are solely those of the authors and do not necessarily represent those of their affiliated organizations, or those of the publisher, the editors and the reviewers. Any product that may be evaluated in this article, or claim that may be made by its manufacturer, is not guaranteed or endorsed by the publisher.

## Footnotes

^1^ https://www.bioinformatics.babraham.ac.uk/projects/fastqc/

^2^ https://itol.embl.de/

^3^ https://github.com/AbeelLab/quanttb

^4^ https://github.com/farhat-lab/fast-lineage-caller

^5^ https://jodyphelan.gitbook.io/tb-profiler/
