# Supplementary material for: Endogenous relapse and exogenous reinfection in recurrent pulmonary tuberculosis: A retrospective study revealed by whole genome sequencing
Source: Front Microbiol. 2023 Feb 17;14:1115295. doi: 10.3389/fmicb.2023.1115295 (PMC9981662; doi:10.3389/fmicb.2023.1115295)
Supplement: Supplementary file 1 [file Data_Sheet_1.PDF]

Supplementary Material

1.1 Supplementary Figures

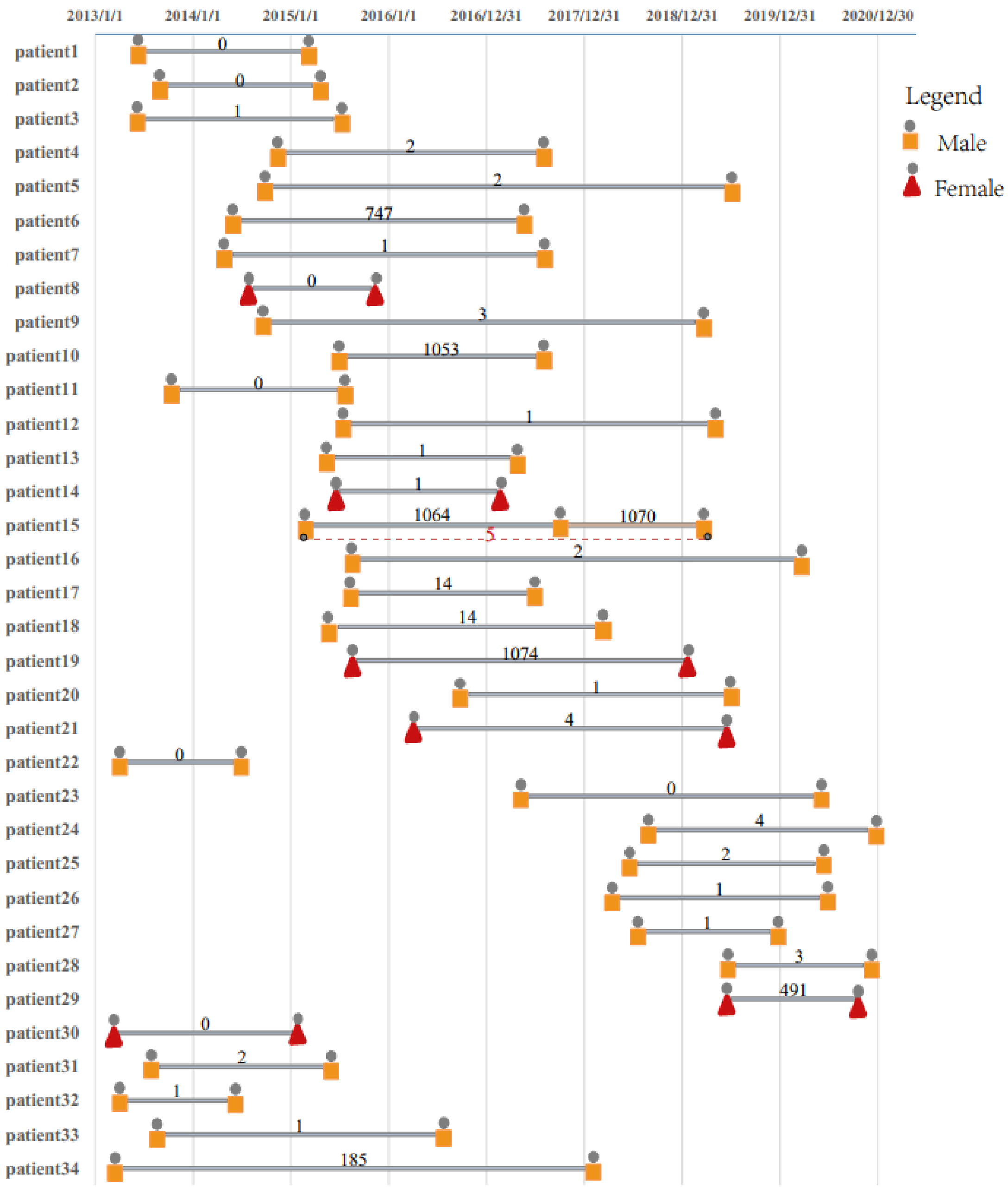

Figure 1. Time interval and SNP distance between initial and TB recurrence

Note: The SNP distances between each pair are indicated on the bars.

1.2 Supplementary Tables

Table S1 SNPs identified between two consecutive isolates in TB relapse

Patient ID	Position	Locus	Gene	Type	Product	Category
patient3	55549	Rv0050	ponA1	INDEL	bifunctional penicillin-insensitive transglycosylase/penicillin-sensitive transpeptidase	Cell wall and cell processes
patient4	1589735	Rv1413		NS	hypothetical protein	Conserved hypotheticals
	2113894	Rv1866		NS	hypothetical protein	Lipid metabolism
patient5	55549	Rv0050	ponA1	INDEL	bifunctional penicillin-insensitive transglycosylase/penicillin-sensitive transpeptidase	Cell wall and cell processes
	3851887	Rv3433c		NS	bifunctional ADP-dependent (S)-NAD(P)H-hydrate dehydratase/NAD(P)H-hydrate epimerase	Conserved hypotheticals
patient7	3188627	Rv2877c		NS	integral membrane protein	Cell wall and cell processes
patient9	243833	Rv0205		S	transmembrane protein	Cell wall and cell processes
	462739	Rv0385		NS	monooxygenase	Intermediary metabolism and respiration
	3129151	Rv2822c		NS	CRISPR type III-associated protein Csm2	Conserved hypotheticals

patient12	4363678	Rv3883c	mycP1	S	membrane-anchored mycosin	Intermediary metabolism and respiration
patient13	55549	Rv0050	ponA1	INDEL	bifunctional penicillin-insensitive transglycosylase/penicillin-sensitive transpeptidase	Cell wall and cell processes
patient14	1497085	Rv1328	glgP	NS	glycogen phosphorylase	Intermediary metabolism and respiration
patient15	759979	Rv0667	rpoB	NS	DNA-directed RNA polymerase subunit beta	Information pathways
	1472991	Rvnr01	rrs	non-coding region	16S ribosomal RNA	
	3014719	Rv2699c		NS	hypothetical protein	Conserved hypotheticals
	3420842	Rv3059	cyp136	NS	cytochrome P450 Cyp136	Intermediary metabolism and respiration
	4247503	Rv3795	embB	NS	arabinosyltransferase B	Cell wall and cell processes
patient16	761101	Rv0667	rpoB	NS	DNA-directed RNA polymerase subunit beta	Information pathways
	1622895	Rv1443c-Rv1444c		intergenic region		
patient20	3325415	Rv2970c	lipN	INDEL	lipase/esterase LipN	Intermediary metabolism and respiration
patient21	6742	Rv0005	gyrB	NS	DNA gyrase subunit B	Information pathways
	7581	Rv0006	gyrA	NS	DNA gyrase subunit A	Information pathways

	1421053	Rv1272c		NS	drug ABC transporter ATP-binding protein	Cell wall and cell processes
	4247431	Rv3795	embB	NS	arabinosyltransferase B	Cell wall and cell processes
patient24	725190	Rv0631c	recC	S	exonuclease V subunit gamma RecC	Information pathways
	796969	Rv0697		NS	dehydrogenase	Intermediary metabolism and respiration
	2520845	Rv2247	accD6	NS	acetyl-/propionyl-CoA carboxylase subunit beta	Lipid metabolism
	3851887	Rv3433c		NS	bifunctional ADP-dependent (S)-NAD(P)H-hydrate dehydratase/NAD(P)H-hydrate epimerase	Conserved hypotheticals
patient25	55549	Rv0050	ponA1	INDEL	bifunctional penicillin-insensitive transglycosylase/penicillin-sensitive transpeptidase	Cell wall and cell processes
	163705	Rv0136	cyp138	NS	cytochrome P450 Cyp138	Intermediary metabolism and respiration
patient26	725190	Rv0631c	recC	S	exonuclease V subunit gamma RecC	Information pathways
patient27	1457144	Rv1300	hemK	NS	release factor glutamine methyltransferase	Intermediary metabolism and respiration
patient28	459399	Rv0383c-Rv0384c		intergenic region		
	1457144	Rv1300	hemK	NS	release factor glutamine methyltransferase	Intermediary metabolism and respiration

	1981605	Rv1752-Rv1753c		intergenic region		
patient31	2449707	Rv2187	fadD15	S	long-chain-fatty-acid--CoA ligase FadD15	Lipid metabolism
	2736027	Rv2438c	nadE	NS	glutamine-dependent NAD(+) synthetase	Intermediary metabolism and respiration
patient32	55549	Rv0050	ponA1	INDEL	bifunctional penicillin-insensitive transglycosylase/penicillin-sensitive transpeptidase	Cell wall and cell processes
patient33	2453420	Rv2190c-Rv2191		intergenic region		
